# Co-Partnering With Older Adults in Qualitative Research

**DOI:** 10.1093/geroni/igaf122.1060

**Published:** 2025-12-31

**Authors:** Joyce Weil, Jarmin Yeh, Theresa Allison

## Abstract

Qualitative research with older adults is a dynamic, interactive process, enriched by rich, thick descriptions and inclusion of participants’ words and images. Traditional research often separates the roles of “researcher” and “participant,” creating power imbalances and limiting participant influence. By co-partnering with older adults at all stages—from planning and conceptualization to data collection, analysis, and dissemination—a more collaborative and reflexive process is achieved, with researchers no longer the sole “experts.” This symposium challenges hierarchical distinctions between “researchers” and “participants” by examining methodologies distributing power and expertise among all stakeholders. We will present 1) a comprehensive model of researcher-participant partnership developed through a virtual photovoice study with older adults, detailing collaborative techniques throughout the research phases; 2) the history and key contributions of a Resource Center on Minority Aging Research, focusing on its Community Liaison and Recruitment Core’s community-engaged health events in Detroit and Flint, Michigan, offering a conceptual model for advancing the science of inclusion; 3) a series of team-based photovoice projects that bridge the gap between academia and community, fostering meaningful collaborations, and informing policy through the lived experiences of diverse aging populations; and 4) findings from a participatory photovoice project documenting the lived experiences of older adults aging in place within San Francisco’s single-room occupancy hotels. This symposium offers theoretical frameworks and practical strategies for reimagining power relationships in gerontological research. By centering older adults’ expertise throughout the research process, these approaches generate more authentic knowledge and create pathways for meaningful community engagement and policy impact. Qualitative Research Interest Group Sponsored Symposium

